# Ovarian Cancer-Driven Mesothelial-to-Mesenchymal Transition is Triggered by the Endothelin-1/β-arr1 Axis

**DOI:** 10.3389/fcell.2021.764375

**Published:** 2021-12-01

**Authors:** Danila Del Rio, Ilenia Masi, Valentina Caprara, Francesca Spadaro, Flavia Ottavi, Raffaele Strippoli, Pilar Sandoval, Manuel López-Cabrera, Ricardo Sainz de la Cuesta, Anna Bagnato, Laura Rosanò

**Affiliations:** ^1^ Institute of Molecular Biology and Pathology, CNR, Rome, Italy; ^2^ Unit of Preclinical Models and New Therapeutic Agents, IRCCS - Regina Elena National Cancer Institute, Rome, Italy; ^3^ Confocal Microscopy Unit, Core Facilities, Istituto Superiore di Sanità, Rome, Italy; ^4^ Department of Molecular Medicine, Sapienza University of Rome, Rome, Italy; ^5^ Centro de Biología Molecular “Severo Ochoa” (CBM), Spanish Council for Scientific Research (CSIC), Universidad Autónoma de Madrid (UAM), Madrid, Spain; ^6^ Department of Gynecoloy and Obstretics, Quirónsalud University Hospital, Madrid, Spain

**Keywords:** mesothelial cells, endothelin-1, serous ovarian cancer, β-arrestin1, mesothelial-to-mesenchymal transition

## Abstract

Transcoelomic spread of serous ovarian cancer (SOC) results from the cooperative interactions between cancer and host components. Tumor-derived factors might allow the conversion of mesothelial cells (MCs) into tumor-associated MCs, providing a favorable environment for SOC cell dissemination. However, factors and molecular mechanisms involved in this process are largely unexplored. Here we investigated the tumor-related endothelin-1 (ET-1) as an inducer of changes in MCs supporting SOC progression. Here, we report a significant production of ET-1 from MCs associated with the expression of its cognate receptors, ET_A_ and ET_B_, along with the protein β-arrestin1. ET-1 triggers MC proliferation via β-arrestin1-dependent MAPK and NF-kB pathways and increases the release of cancer-related factors. The ET_A_/ET_B_ receptor activation supports the genetic reprogramming of mesothelial-to-mesenchymal transition (MMT), with upregulation of mesenchymal markers, as fibronectin, α-SMA, N-cadherin and vimentin, NF-kB-dependent Snail transcriptional activity and downregulation of E-cadherin and ZO-1, allowing to enhanced MC migration and invasion, and SOC transmesothelial migration. These effects are impaired by either blockade of ET_A_R and ET_B_R or by β-arrestin1 silencing. Notably, in peritoneal metastases both ET_A_R and ET_B_R are co-expressed with MMT markers compared to normal control peritoneum. Collectively, our report shows that the ET-1 axis may contribute to the early stage of SOC progression by modulating MC pro-metastatic behaviour via MMT.

## Introduction

The spreading of serous ovarian cancer (SOC) cells through the peritoneal cavity and the metastasis to the omentum and peritoneum is a common characteristic in patients with advanced tumor (Lengyel, 2010; Yeung et al., 2015). In this tumor microenvironment (TME), the complex and bidirectional interactions among SOC, immune and stromal cells, act as intrinsic features of the tumor supporting metastatic colonization, thus emphasizing the need to gain a comprehensive understanding of the mechanisms regulating cell-cell interactions (Naora and Montell, 2005). SOC cells exfoliate as single cells or multicellular spheroids that disseminate with the peritoneal fluid flow (Ahmed and Stenvers, 2013; Worzfeld et al., 2017; Rickard et al., 2021). Metastatic spread is largely mediated by the ability of spheroids, but also individual cells, to adhere to a monolayer of peritoneal mesothelial cells (MCs) resting upon a basement membrane, to induce their retraction and to penetrate the sub-mesothelial interstitial collagen-rich extracellular matrix (ECM), followed by their survival, and tumor nodule formation with surrounding host cells (Burleson et al., 2004; Shield et al., 2009; Mogi et al., 2021). In this context, the invasive behaviour of SOC spheroids depends upon their contractile capacity and involves both integrins and cadherins (Sodek et al., 2009; Iwanicki et al., 2011; Klymenko et al., 2017a; Klymenko et al., 2017b; SacksSuarez et al., 2019; Goyeneche et al., 2020). Moreover, the presence of N-cadherin cell-cell junctions in ascitic spheroids as well as matrix metalloprotease (MMP)-mediated proteolysis are key determinants in mesothelial clearance and matrix invasion, thus predicting more metastatic lesions (Klymenko et al., 2017a; Klymenko et al., 2017b).

Although for many years mesothelial tissue has been considered as a sheet of cells with low mobility, serving as a barrier against tumors, recent studies demonstrated that they can be reprogrammed by TME to acquire invasive properties and tumor-supporting functions, mainly in the advanced stages of SOC tumor, which is likely to develop peritoneal dissemination (Rynne-Vidal et al., 2015; Mogi et al., 2021). MCs might be altered by SOC cells through the stimulation of soluble factors present in ascites (Matte et al., 2014; Matte et al., 2019). In turn, MCs strongly contribute to peritoneal environment alteration in terms of direct and indirect cell-to-cell crosstalk with tumor cells (Matte et al., 2016). SOC cells can attach directly to MCs through upregulation of integrins and CD44 (Lessan et al., 1999), and MCs might support the adhesion and invasion of SOC cells through ligands, such as collagen and fibronectin, thus contributing to ECM remodelling (Kenny et al., 2014; Natarajan et al., 2019). On the other hand, SOC cells upregulate matrix proteases to cleave ECM proteins, thus providing a further adhesion of cancer cells to MCs, after which SOC cells can invade the submesothelial matrix (Kenny et al., 2011). In addition, increasing evidence demonstrates the existence of a parallel bidirectional signaling and nutrient exchange between cancer and mesothelial cells (Hart et al., 2019). Recent findings also support the role of MCs in the heterotypic of spheroids in malignant ovarian cancer ascites (Shishido et al., 2018; Matte et al., 2019).

MCs, which are mesodermal in origin and possess both epithelial and mesenchymal features, are linked by several types of intercellular junctions, including tight junctions (TJs), adherens junctions, desmosomes and gap junctions (Kastelein et al., 2019). SOC cells may disrupt the intercellular junctions within the MCs, favouring transmigration through the mesothelial monolayer (Pakula et al., 2019a). In this context, during the metastasis process, a subset of MCs undergoes a mesothelial-to-mesenchymal transition (MMT), a complex and step-wise process that begins with alterations in cellular architecture and include a deep molecular reprogramming with new biochemical instructions (Pakula et al., 2019b). Commonly used molecular markers for MMT include the downregulation of cytokeratins, adherens junction protein E-cadherin and calretinin, and the up-regulation of N-cadherin and transcription factor Snail (Lopez-Cabrera, 2014). In this process, MCs adopt a front to back polarity and acquire α-SMA expression, with increased capacity to migrate and to invade the submesothelial space, affecting the peritoneal niche, including matrix remodelling and angiogenesis (Lopez-Cabrera, 2014; Rynne-Vidal et al., 2015; Pakula et al., 2019b). This transformation can be mediated by several soluble factors secreted by cancer cells and found in ascites (Lv et al., 2011; Nakamura et al., 2015). All these findings highlight the role of MCs no longer as passive bystanders, but active coordinators of SOC progression.

Within the SOC ascitic fluids, the small peptide endothelin-1 (ET-1) is present together with several other factors, including vascular endothelial growth factor (Salani et al., 2000; Rosanò et al., 2013). The ET family comprises three structurally similar 21-amino acid peptides. ET-1 and -2 activate two G-protein coupled receptors, ET_A_ and ET_B_, with equal affinity, whereas ET-3 has a lower affinity for the ET_A_ subtype. ET-1 is the most abundant isoform in the human cardiovascular system and the primary source is thought to be vascular endothelial cells, although the peptide is produced by other cell types, including epithelial cells, macrophages and monocytes, glial cells and neurons (Davenport et al., 2016; Davenport et al., 2018). The ET-1 axis is most recognized for its potent vasoconstrictive action involved in the physiological regulation of vascular tone. Furthermore, ETs play a role in pathologies such as heart failure, renal insufficiency, septic shock, atherosclerosis, and haemorrhage-associated cerebrovascular conditions, and can worsen insulin resistance by impairing glucose uptake in skeletal muscles (Barton and Yanagisawa, 2019). Chronic endothelin stimulation has been implicated in several human cardiovascular, inflammatory, fibrogenic and oncologic diseases (Torres Crigna et al., 2021).

An active ET-1 axis, involving the interaction with ET_A_R and ET_B_R, supports SOC growth and progression, and key signaling pathways activated by ET-1 related to invasion and metastasis are orchestrated by the cooperation of protein β-arrestins (β-arrs) with signaling proteins in cytosolic or nuclear compartments (Rosanò et al., 2013; Peterson and Luttrell, 2017; Bagnato and Rosanò 2019; Tocci et al., 2019). Our recent studies addressing the question of how the ET-1 axis contributes to the reorganization of the cytoskeleton and cell motility during SOC metastatic progression uncovered a relevant role in invadopodia, F-actin-based protrusive membrane structures operating focused matrix degradation (Masi et al., 2021a). Acting as an anchoring point for players and signaling molecules regulating cell adhesion, invasion and proteolysis, β-arr1 determines the convergence of specific signal transducers (Semprucci et al., 2016; Di Modugno et al., 2018; Chellini et al., 2019). More recently, we provide a direct mechanism by which the ET_A_R/β-arr1 pathway integrates adhesion and proteolytic signaling through integrin-linked kinase (ILK)/Rac3 GTPase operating invadopodia-dependent ECM remodelling and cell invasion (Masi et al., 2021b). Since the molecular mechanisms by which MCs are attracted to and communicate with SOC cells are partially understood, we propose to study ET-1 as a cell-secreted factor promoting MCs activation and recruitment supporting invasive behaviour of SOC cells.

## Materials and Methods

### Patient Biopsies

Paraffinized peritoneal biopsies from 5 SOC were used for immunohistochemical staining. Additionally, peritoneal tissue samples from three non-oncological patients were considered as control. Informed written consent was obtained from the patients, with the approval of the Ethics Committee of Hospital Fundación Jiménez Díaz (Madrid, Spain; ethic approval number: 11/17).

### Cell Lines

Omental derived adult primary mesothelial cells were obtained from Zen-Bio (MCs) (Cary, NC, United States) or Creative Bioarray (MC1s) (Shirley, United States) and cultured in Mesothelial Cell Growth Medium (Cat# MSO-1; Zen-Bio). Human ovarian serous adenocarcinoma cell line SKOV3 was obtained from the American Type Culture Collection (ATCC) (LGC Standards, Teddington, United Kingdom), and maintained in McCoy’s 5A medium (Cat# 26600-023; Thermo Fisher Scientific). OVCAR3 was obtained from ATCC (Manassas, VA) and maintained in RPMI1640 GlutaMAX (Cat# 61870-010; Thermo Fisher Scientific). Human ovarian carcinoma cell line HEY were obtained from Cell Biosystem and cultured in 1X DMEM with 2 mM L-Glutamine. Media were supplemented with 10% fetal calf serum (FCS) (Thermo Fisher Scientific) containing penicillin (10.000 U/ml)-streptomycin (10 mg/ml) (Euroclone). All cell cultures were maintained at 37°C, 5% CO2 and 95% humidity and cells were routinely tested for the absence of viral/bacterial/fungal/mycoplasma contamination.

### Antibodies and Chemical Reagents

Primary antibodies used for Western blotting (WB) were as follows: anti-Endothelin A receptor (Cat# PA3-065; Thermo Fisher Scientific), anti-Endothelin-B receptor (Cat# ab117529; Abcam), anti-β-arr1 (Cat# ab32099; Abcam), anti-Tubulin (Cat# sc-32,293; Santa Cruz), anti-N-cadherin (Cat# 610254; BD Transduction Laboratories), anti-E-cadherin (Cat# 610182, BD Transduction Laboratories), anti-pp42/44 MAPK (Cat# 4370; Cell Signaling), anti-p42/44 MAPK (Cat# 4695; Cell Signaling), anti-p65-NF-kB (Cat# 3031L, Cell Signaling), anti-NF-kB (Cat# 3034, Cell Signaling), anti-Fibronectin (Cat# ab2413, Abcam), anti-GAPDH (Cat# G9543, Sigma Life science), anti-ZO-1(Cat #40-2200, Invitrogen). Secondary antibodies used for WB were as follows: horseradish peroxidase-conjugated rabbit anti-goat or anti-mouse Abs. Primary antibodies used for immunofluorescence were as follows: anti-Fibronectin (Cat# ab2413, Abcam), anti-E-cadherin (Cat# 610182, BD Transduction Laboratories), anti-α-SMA (Cat# NCL-L-SMA, Leica), anti-Snail (Cat# SC10432, Santa Cruz). Secondary antibodies used were as follows: Alexa Fluor 488 phalloidin (Cat# A12379; Thermo Fisher Scientific), Alexa Fluor 488 (Cat# A1101; Thermo Fisher Scientific), Alexa Fluor 594 (Cat# A11037; Thermo Fisher Scientific).

Chemical reagents used were as follows: 4′,6′-diamidino-2-phenykindole (DAPI) (Cat# 1331762; Bio-Rad Laboratories), Vectashield (Cat# H-1000; Vector Laboratories), PKH26 Red Fluorescent Cell Linker Kit for General Cell Membrane Labeling (Cat# PKH26GL-1KT, Sigma-Aldrich), ET-1 (100 nmol/L) (Cat# E7764-1MG; Sigma-Aldrich), BQ788 (1µM) (Peninsula Laboratories), BQ123 (1µM) (Cat# L01435, Alexis Corporation), Ambrisentan (1µM) (Cat# SML2104; Sigma-Aldrich) also called (+) - (2S) - 2-[(4,6dimethylpyrimidin-2-yl) oxy]-3-methoxy-3,3-diphenylpropanoic acid, NF-kB inhibitor Caffeic acid phenethyl ester (20 µM) (Cat# 104594-70-9, Tocris).

### RNA Isolation and Semiquantitative PCR e Quantitative Real Time PCR

Total RNA was extracted from cells using PureZol (Cat# 7326880 BioRad), according to the manufacturer’s instructions and 1 µg was used for retrotranscription (RT) using PrimeScrip RT Reagent Kit (Cat# RR037A, Takara). cDNA was examined by semiquantitative PCR, conducted in the automated DNA Thermal Cycler GeneAmp PCR System 9700 (Applied Biosystem) using AmpliTaq DNA Polymerase (Applied Biosystem). The primers used were as follow:

ET_A_R (EDNRA) F: 5′-GTG​GCT​CTT​CGG​GTT​CTA​TTT-3′

ET_A_R (EDNRA) R: 5′-CGG​TTC​TTG​TCC​ATC​TCG​TTA-3′

ET_B_R (EDNRB) F: 5′-AGT​GGA​TTG​GTG​GGC​ATT​AG-3′

ET_B_R (EDNRB) R: 5′-GGA​CAT​AGG​AGG​AGA​GGA​GAA-3′

β-arr1 (ARRB1) F:5′- GAG​CAC​GCT​CTT​ACC​TTT​CAC-3′

β-arr1 (ARRB1) R:5′-TCTCTGGGGCATACTCTGAACC-3′

Cyclophilin A F: 5′- TTC​ATC​TGC​ACT​GCC​AAG​AC-3′

Cyclophilin A R: 5′-TCG​AGT​TGT​CCA​CAG​TCA​GC-3′

The PCR products were analysed by electrophoresis on 1% agarose gel and visualized by using ChemiDoc Imaging System and ImageLab Software (Bio-Rad Laboratories).

Quantitative real-time-PCR was performed by using the light Cycler QuantStudio 3 qPCR System (Applied Biosystem) using SensiFAST™ SYBR® Hi-ROX One-Step Kit (Meridian Bioscience). The number of each gene-amplified product was normalized to the number of GAPDH amplified products. The primers used were as follow:

ET-1 (EDN1) F: 5′-GTG​TCT​ACT​TCT​GCC​ACC​TG-3′

ET-1 (EDN1) R:5′-AAGTAAATTCTCAAGGCTCTCT-3′

GAPDH F:5′-ACATCGCTCAGACACCATG-3

GAPDH R:5′-TGTAGTTGAGGTCAATGAAGGGG-3′

### Silencing of β-arr1

Silencing of β-arr1 was performed using ON-TARGET plus SMART pool siRNAs (L-011971-00) for 48 h and siGENOME Control Pool Non-targeting was used as a negative control (SCR) (Dharmacon) (Di Modugno et al., 2018). In brief, 1 × 10^5^ cells were seeded and cultured in six-well plates until they reached 70–80% confluence and transiently transfected for 48 h, using Lipofectamine RNAiMAX (Cat# 13778; Invitrogen) reagent, according to the manufacturer’s instructions. The total cell lysates were collected and analyzed by WB to confirm efficient knockdown.

### Luciferase Reporter Gene Assay

To measure the NF-κB promoter activity, cells were transiently co-transfected with a 1 μg NFκB-Luc vector that contains multiple copies of the NF-κB consensus sequence fused to a TATA-like promoter (PTAL) or with the negative control, the pTAL-Luc vector. To measure the transcriptional activity of SNAIL, we used Snail_pGL2, a gift from Paul Wade (Addgene plasmid # 31694; http://n2t.net/addgene:31694;RRID:Addgene_31694) (Fujita et al., 2003). To measure the transcriptional activity of E-cadherin promoter cells were transiently transfected with 1 μg pGL2 Ecad3/Luc construct (kindly provided by Dr E.R. Fearon, University of Michigan, Ann Arbor, MI), or with empty control vectors (Promega). Cells in different experimental conditions were plated in a six well plate until they reached 70–80% confluence and transiently cotransfected with indicated plasmids together with the control the pCMV-β-galactosidase vector (Promega) using Lipofectamine 2000 reagent (Invitrogen). Serum-free medium alone or with ET-1 and/or BQ123+BQ788, and with NF-kB inhibitor and/or ET-1 were added to the wells and incubated for an additional 24 h. Reporter activity was measured by using the Luciferase assay system (Cat# E1500, Promega) quantified by using a microplate reader (CLARIOstar, BMG Labtech). Luciferase activities were normalized to β-galactosidase activity by using a microplate reader (Neo Biotech). The mean of three independent experiments performed in sextuplicate was reported.

### Western Blotting

For WB analysis, cells were detached by scraping, collected by centrifugation, and lysed in RIPA buffer [50 mMTris·HCL (pH 7.5), 150 mm NaCl, 1% Nonidet P-40, 0.5% sodium deoxycholate (NaDoc), 0.1% SDS] containing proteases and phosphatase inhibitors (Roche). Protein concentrations were determined using the DC Protein assay (Bio-Rad Laboratories). Cell lysates were resolved on MiniPROTEAN TGX gels and transferred to nitrocellulose membranes (Bio-Rad Laboratories), followed by WB using the primary antibodies. Primary antibodies were revealed using horseradish peroxidase-conjugated goat anti-rabbit or anti-mouse Abs (Bio-Rad Laboratories). Proteins were visualized by chemiluminescence (Clarity Western ECL Substrates, Bio-Rad Laboratories) by using Azure 300 (Azure Biosystems). Quantification analyses were performed by ImageJ (https://imagej.nih.gov/ij/), a Java-based freeware, and reflects the relative amounts as a ratio of each protein band relative to the lane’s loading control.

### Human Oncology Array

A Human Oncology Array Kit (ARY026) was purchased from R&D (Minneapolis, United States). Briefly, the conditioned medium from untreated or ET-1-treated MCs (72 h) was added to each antibody-printed nitrocellulose membrane for overnight incubation at 4°C, following the manufacturer’s instructions. The membranes were visualized by using ChemiDoc Imaging System. Each pair of positive dots represented signals of highly expressed cytokines and the intensity was quantified by ImageJ software. The full list of all proteins candidates is available at the manufacturer’s official website (Proteome Profiler Human XL Oncology Array ARY026: R&D Systems (rndsystems.com).

### ELISA

Following the manufacturer’s protocols, uPAR, MMP-7 and ET-1 secretion were evaluated by using a Human uPAR Immunoassay (Cat# DUP00 R&D), Human Total MMP-7 Quantikine ELISA Kit (Cat# DMP700, R&D), and ET-1 Human Endothelin 1 ELISA Kit (Car# CEK1146, Cohesion Biosciences), respectively, following manufacturer’s instructions. The concentration of each desired protein in each sample was determined by interpolating the absorbance values against the standard curve that was calculated by recombinant proteins at gradient dilution.

### Immunofluorescence and Confocal Laser Scanning Microscopy

Cells cultured on coverslips were fixed with 4% paraformaldehyde for 10 min at room temperature, permeabilized with 0.2% Triton-X-100 and blocked with 0.1 M glycine, 1% BSA and 0.1% Tween20 in PBS for 30 min at room temperature. Samples were incubated with primary Abs in 0.5% BSA in PBS overnight at 4°C, followed by incubation with secondary Abs conjugated with Alexa Fluor 488 (Cat# A11001; Thermo Fisher Scientific) and Alexa Fluor 594 (Cat# A11037; Thermo Fisher Scientific) for 1h at room temperature. Actin cytoskeleton was visualized by using Alexa Fluor 633 phalloidin (Cat# A22284; Thermo Fisher Scientific). Nuclei were stained with DAPI. Coverslips were finally mounted with a Vectashield mounting medium for fluorescence (Vector Laboratories). CLSM observations were performed with a Zeiss LSM980 apparatus, using a 63×/1.40 NA oil objective and excitation spectral laser lines at 405, 488, 543, 594, and 639 nm. Image acquisition and processing were carried out using the Zeiss Confocal Software Zen 3.1 (Blue edition). Signals from different fluorescent probes were taken in sequential scan settings and co-localization were visualized in merge images.

### Cell Viability

MCs (3 × 10^4^) were cultured on a 24-well plate in different experimental conditions. Serum-free medium alone or with ET-1 and/or BQ123+BQ788 or conditioned media (CM) from SKOV3 or SKOV3 treated with AMB or from HEY or HEY treated with AMB was added and left for different times (24, 48, 72 h) at 37°C. Total cells from each well were then recuperated by using Trypsin-EDTA 1X in PBS 100 ML (Euroclone) solution and counted by using an automated cell counter (Beckman Coulter). The experiment was performed in triplicates for all conditions described and repeated at least three times. As indicated, we used Cyto3DTM Live-Dead Assay Kit (TheWell Bioscience, Inc., North Brunswick, NJ, United States) to determine the live/dead nucleated cells by using a dual-fluorescence system of Acridine orange (AO) and propidium iodide (PI), both nuclear staining (nucleic acid binding) dyes. All live nucleated cells fluoresce green, and all dead nucleated cells fluoresce red. Several images were taken by using Bio-Rad ZOE fluorescent cell image under a phase-contrast microscope (Bio-Rad Laboratories).

### Wound-Healing Assay

The assays were carried out seeding MCs (3 × 10^5^ cells per well) in a culture-Insert 2 Well in µ-Dish 35 mm (Cat# 81176, IBIDI). Cells in different cell conditions were coloured with PKH26 and seeded into the insert. Once the insert was removed, cells were treated with ET-1 and/or BQ123+BQ788 or CM from SKOV3 or SKOV3 treated with AMB or from HEY or HEY treated with AMB and were left to migrate at 37°C until the open area was closed in at least one experimental group. Each experiment was performed in triplicates for all conditions described and repeated at least three times. From each Dish, several images were taken by using Bio-Rad ZOE fluorescent cell imager under a phase-contrast microscope. Quantification analyses were performed by ImageJ by measuring the opened area of each experimental group versus the open area of the control group.

### Transwell Invasion Assay

The assays were carried out using an insert 8.0 µm pore sized membranes (Cat# 662638; Greiner Bio-one). MCs (3 × 10^4^), in different experimental conditions, were stimulated with serum-free medium alone or with ET-1 and/or BQ123+BQ788, or CM from SKOV3 or SKOV3 treated with AMB or from HEY or HEY treated with AMB, added to the lower chamber precoated with Cultrex Basement Membrane Matrix (Cat# 3500-096-03; Trevigen). The cells were left to migrate for 12 h at 37°C. Cells on the upper part of the membrane were scraped using a cotton swab, and the migrated cells were stained using Three-Step Stain Set (Cat# 3300; Thermo Fisher Scientific). Each experiment was performed in triplicates for all conditions described and repeated at least three times. From each transwell, several images were taken by using Bio-Rad ZOE fluorescent cell imager under a phase-contrast microscope, and four broad fields were considered for quantification.

### Transmesothelial Migration Assay

MCs (1 × 10^5^) were seeded in 8.0 µm pore sized membranes (Cat# 662638; Greiner Bio-one) coated with fibronectin (10 µg/ml) (Cat# F2518-5MG; Sigma-Aldrich) and left to form a monolayer for 48 h at 37°C. OVCAR3 cells (30 × 10^3^), in different experimental conditions, were stained with PKH26 for 5 min at 37°C, washed with a complete medium, plated onto a mesothelial monolayer, and allowed to migrate for 12 h. Serum-free medium alone or with ET-1 and/or BQ123+BQ788 were added to the lower chamber. Transmigrated cells were photographed by using Bio-Rad ZOE fluorescent cell imager and the results of the analysis of the individual photos are reported, normalized to control and shown as a fold of control. The experiment was performed in triplicates for all conditions described and repeated at least three times.

### Immunohistochemistry

Immunohistochemical analysis (IHC) was performed on serial sections 3 μm thick. Deparaffinized tissues were heated to expose the hidden antigens using Antigen Retriever containing citrate buffer, pH 6.0 (Sigma Aldrich, St. Louise, United States). Endogenous peroxidase was blocked with Real Peroxidase-Blocking Solution (Dako, Glostrup, Denmark). Samples were stained using primary antibodies to detect podoplanin (Origene Technologies, Rockville, United States); *α*-SMA (Sigma Aldrich); ET_A_R (ThermoFisher Scientific) and ET_B_R (Abcam, Cambridge, United Kingdom). A biotinylated secondary antibody (Vector Laboratories, Burlingame, CA, United States) followed by R.T.U Vectastain Elite ABC Kit (Vector Laboratories) was applied to detect primary antibodies. All cases were revealed using DAB (Dako) as chromogen and finally counterstained with haematoxylin. Representative images were captured with a digital camera coupled to a brightfield microscope.

### Statistical Analysis

All the experiments were repeated at least three times, otherwise indicated. Statistical analysis was conducted using GraphPad Prism software and the values represent mean ± SD. Graphs comparing two conditions were analyzed via unpaired *t*-test with Welch’s correction. Graphs comparing more than two conditions were analyzed via one-way ANOVA followed by Tukey correction for multiple comparisons. Statistical significance was defined as *p < 0.05; **p < 0.01; ***p < 0.001; ****p < 0.0001.

## Results

### Mesothelial Cells Express ET-1 Receptors and Secrete ET-1, Promoting Cell Proliferation

**FIGURE 1:**
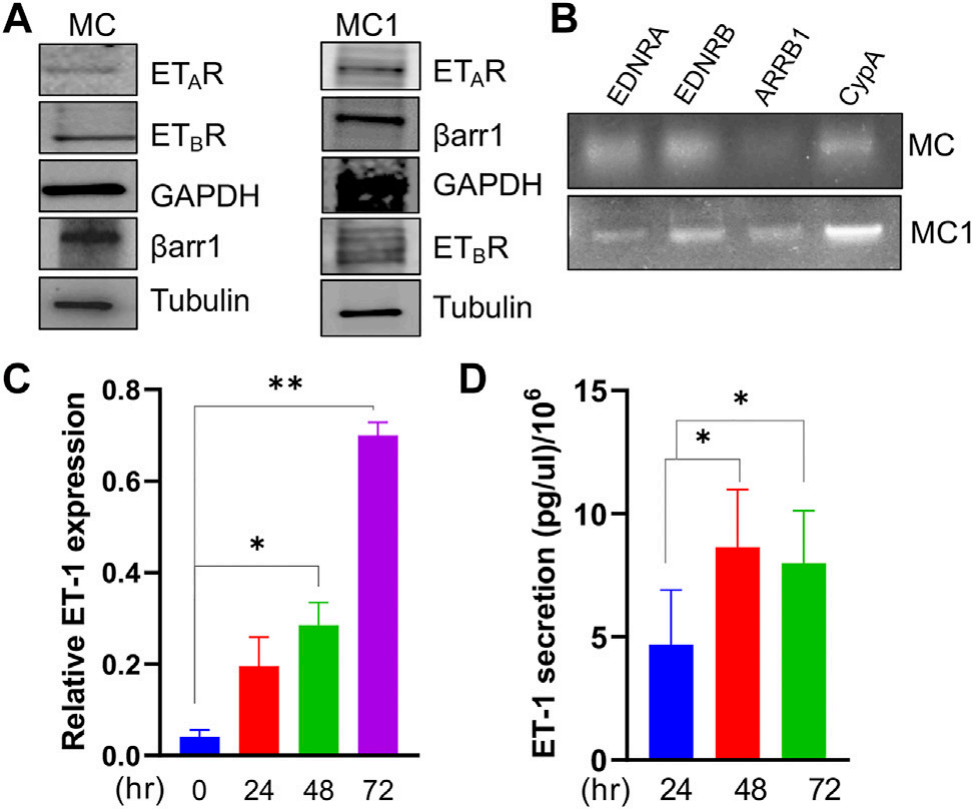
Mesothelial cells express ET_A_R and ET_B_R along with β-arr1 and secrete ET-1. **(A)** Representative Western Blotting (WB) analysis of ET_A_R and ET_B_R and β-arr1 in MCs and MC1s. GAPDH and Tubulin were used as the loading control. **(B)** Representative RT-PCR analysis showing ET_A_R, ET_B_R and β-arr1 mRNA levels in MCs. CypA was used as a loading control. **(C)** ET-1 expression evaluated by qPCR in MCs at 24, 48, and 72 h **(D)** ET-1 secretion evaluated by ELISA assays in MC culture medium at 24, 48, and 72 h. Histograms, the mean ± SD, *n* = 2. One-way ANOVA.

Considering ET-1 as a factor influencing the activity of tumor and stromal cells and the importance of MCs to SOC progression (Rosanò et al., 2013), we set out to investigate ET-1 as a candidate affecting MC behaviour and activation. We first examined the expression of ET-1 and its receptors in human primary MCs. These cells express constitutively ET-1 as well as both ET_A_R and ET_B_R, although to a different extent, along with β-arr1, at mRNA and protein levels (Figures 1A–C). The expression of ET-1 mRNA enhances between 24 and 72 h of serum-free culture (Figure 1C). Moreover, they secrete ET-1 in their conditioned media reaching the level of 8 pg/µl/10^6^ cells between 48 and 72 h (Figure 1D).

**FIGURE 2:**
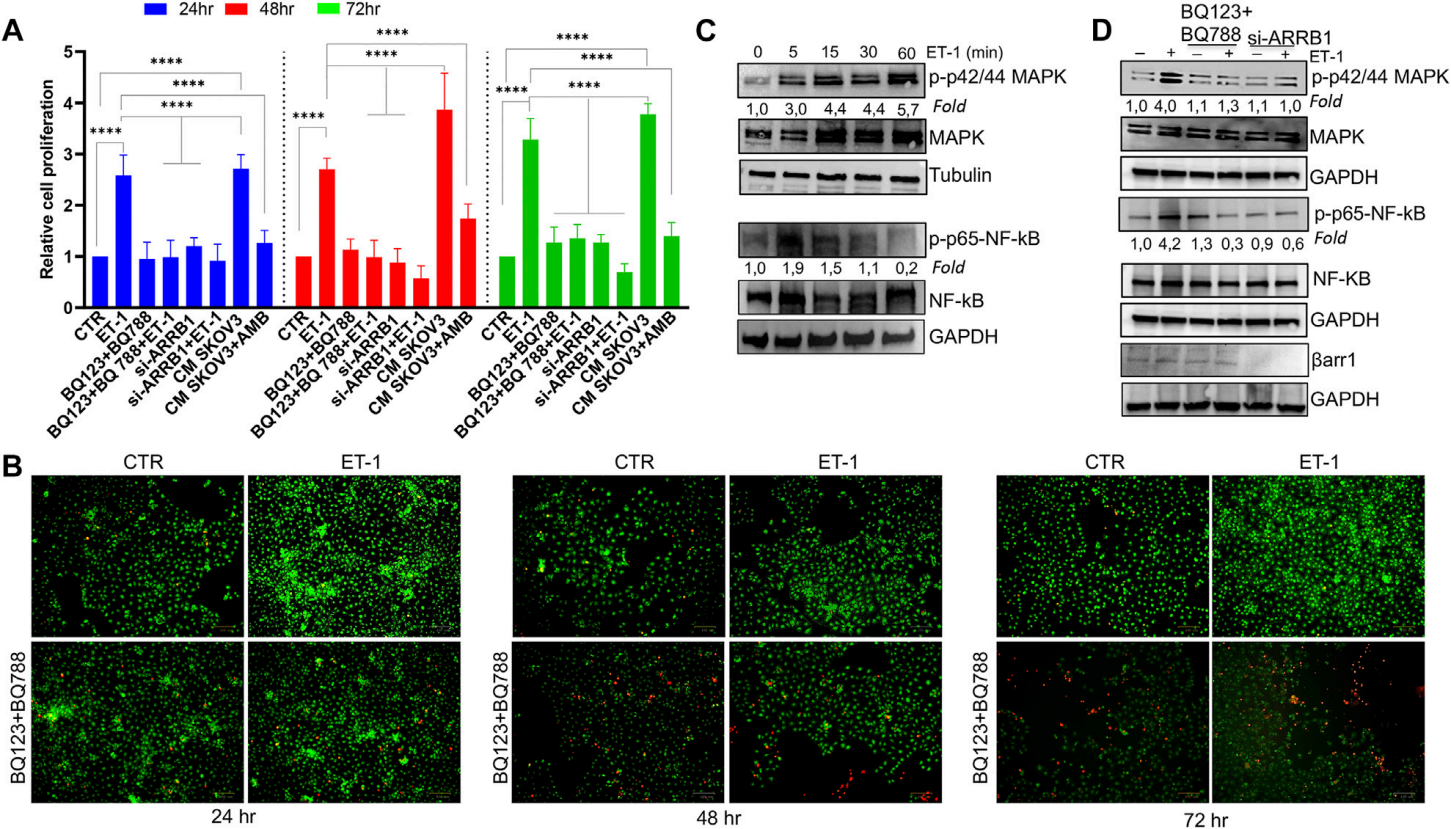
ET-1 enhances MC proliferation through ET_A_R and ET_B_R and βarr1. **(A)** The relative proliferation of MCs untransfected or transfected, when indicated, with si-ARRB1 and treated with ET-1 and/or BQ123+BQ788 or with conditioned medium (CM) from SKOV3 untreated or treated with AMB at indicated time points. Histogram, the mean ± SD, *n* = 6. One-way ANOVA. **(B)** Live-Dead images of MCs after ET-1 and/or BQ123+BQ788 treatment for the indicated times. Green stain, live cells; red stain, dead cells. Scale bar, 100 μm. **(C)** Representative WB analysis of p-p42/44 MAPK/p42/44 MAPK and p-p65-NF-kβ/p65-NF-kβ levels in MCs stimulated or not with ET-1 for the indicated times. **(D)** Untransfected or si-ARRB1-transfected MCs stimulated with ET-1 and/or BQ123+BQ788 for 5 min were subjected to WB analysis for p-p42/44 MAPK/p42/44 MAPK and p-p65-NF-kβ/p65-NF-kβ levels. GAPDH or tubulin was used as the loading control. Phosphoproteins were normalized to the respective total proteins and indicated as fold-over CTR.

According to the well-known role of ET-1 as a mitogenic factor for cancer and stromal cells (Rosanò et al., 2013; Caprara et al., 2014; Tocci et al., 2019), the addition of exogenous ET-1 increases cell proliferation between 24 and 72 h, while the addition of both the ET_A_R and ET_B_R antagonists, BQ123 and BQ788, respectively, completely blocked ET-1 effects, supporting the presence of an ET-1 autocrine loop involving both receptors (Figure 2A). Previous studies showed the role of β-arr1 in ET-1-dependent cell proliferation (Rosanò et al., 2013; Rosanò et al., 2014; Cianfrocca et al., 2017; Tocci et al., 2019). Since MCs also express β-arr1 (Figure 1A), we evaluated its involvement in cell proliferation. As shown in Figure 2A, silencing of β-arr1 significantly reduces ET-1-dependent effect, demonstrating that ET-1/ET-1 receptors/β-arr1 axis controls MC proliferation. To evaluate whether a paracrine ET-1 secreted by SOC cells can also act as a regulator of MCs, we tested the effect of conditioned media (CM) from SKOV3 or HEY cells untreated or treated with the ET_A_R antagonist Ambrisentan (AMB). A significant proliferative effect is evident in cells cultured with control CM, while this effect is absent when CM from AMB-treated cells was used (Figure 2A; Supplementary Figure S1A), supporting the role of cancer cell-derived ET-1 as a factor affecting MC proliferation. According to cell viability analysis, a lower number of live cells is evident after BQ123+BQ788 treatment, compared to ET-1, between 24 and 72 h, reaching a maximum after 72 h of treatment, by using Live-Dead staining assays, further providing evidence for the inhibitory activity of BQ123+BQ788 treatment (Figure 2B).

Since the MAPK, as well as NF-kB cascade, regulate cell growth in cancer (Gilmore 2006; Ullah et al., 2021), the effect of ET-1 on these signaling pathways was evaluated. Time-dependent activation of p42/44 MAPK and NF-kB p65 phosphorylation occurs in response to ET-1 stimulation, compared to control cells, until 30–60 min of ET-1 addition (Figure 2C). The presence of both BQ123 and BQ788 or the silencing of β-arr1 reduces ET-1-dependent phosphorylation levels (Figure 2D), confirming that ET-1 receptors/β-arr1 regulate these pathways.

### ET-1 Acts as a Regulator of Mesothelial-to-Mesenchymal Transition

**FIGURE 3:**
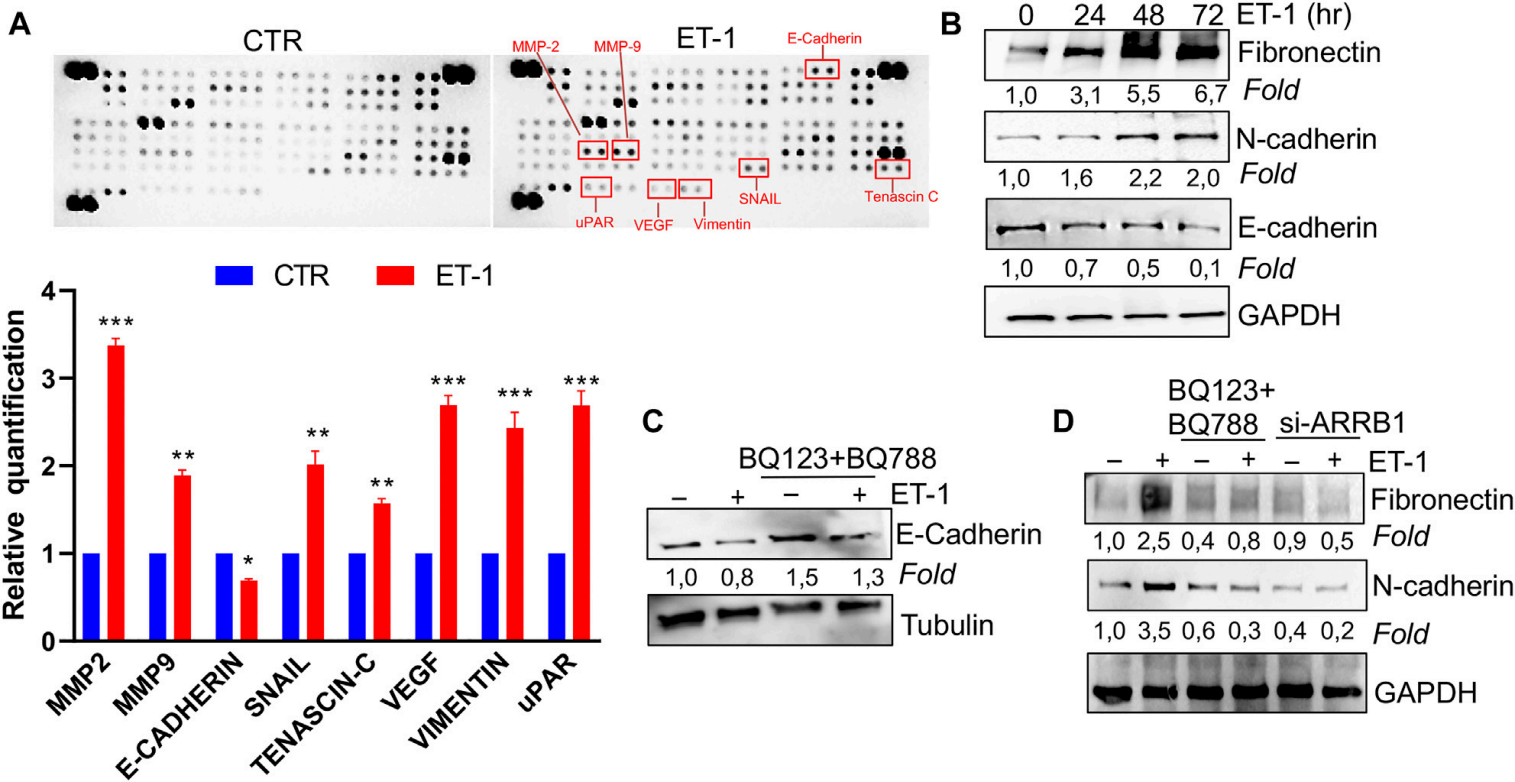
ET-1 regulates MMT marker expression through ET_A_R and ET_B_R/β-arr1 axis. **(A)** Detection of multiple analytes in CM from untreated or ET-1-treated cells (72 h) by using proteome profiler array. The graph summarizes the relative signal intensity of indicated molecules and is shown as fold-over CTR. Histograms, the mean ± SD, *n* = 2. One-way ANOVA. **(B)** Representative WB analysis of indicated proteins in MCs stimulated with ET-1 at indicated times. **(C)** Representative WB analysis of E-Cadherin in MCs stimulated with ET-1 and/or BQ123+BQ788 at 48 h **(D)** MCs transfected with si-ARRB1and stimulated by ET-1 and/or BQ123+BQ788 for 48 h. Proteins were normalized in comparison with the GAPDH or Tubulin, used as the loading control, and indicated as fold-over CTR.

We next assessed the possibility that ET-1 addition in MCs may result in the secretion of factors promoting tumor cell growth and progression. For this purpose, proteome profiler arrays were employed allowing for simultaneous detection of 84 human cancer-related proteins in CM of MCs unstimulated, as control, or stimulated with ET-1 for 72 h. Enhanced levels of many cancer-related proteins are found in the CM of ET-1-treated cells, including MMPs, Snail, Tenascin-C, Vimentin, and downregulated levels of E-cadherin (Figure 3A; Supplementary Figure S1B). Since many of these proteins are related to MMT conversion, the changes in the levels of MMT-related proteins, E-cadherin and ZO-1, typical epithelial markers, and N-cadherin and Fibronectin, typical mesothelial markers, were evaluated by Western Blot analysis. Upregulation of Fibronectin and N-cadherin is observed in ET-1-stimulated cells, associated with a reduced expression of E-cadherin, with a maximum between 48 and 72 h of ET-1 treatment (Figure 3B), while these effects are reverted by the pre-treatment with both BQ123 and BQ788 as well as by after the silencing of β-arr1 (Figures 3C,D).

**FIGURE 4:**
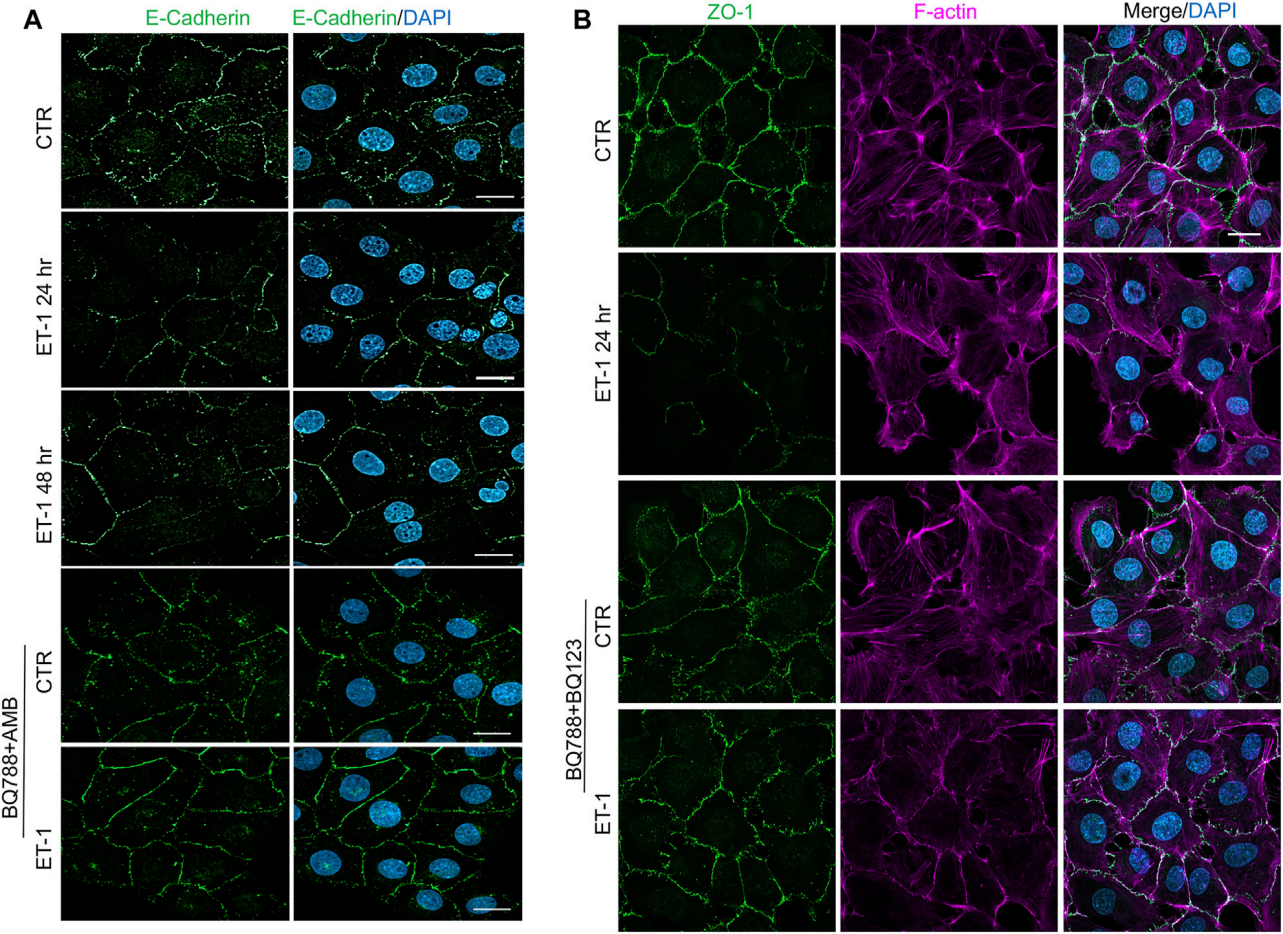
ET-1 induces loss of E-cadherin and ZO-1 through ET_A_R/ET_B_R. **(A)** Representative images of IF analysis of MCs stimulated with ET-1 and/or BQ788+AMB for 48 h. Cells are stained for E-Cadherin (green), and DAPI (blue). **(B)** IF analysis of MCs stimulated with ET-1 and/or BQ123+BQ788 for 24 h. Cells were stained for F-actin (violet), ZO-1 (green) and DAPI (blue).

**FIGURE 5:**
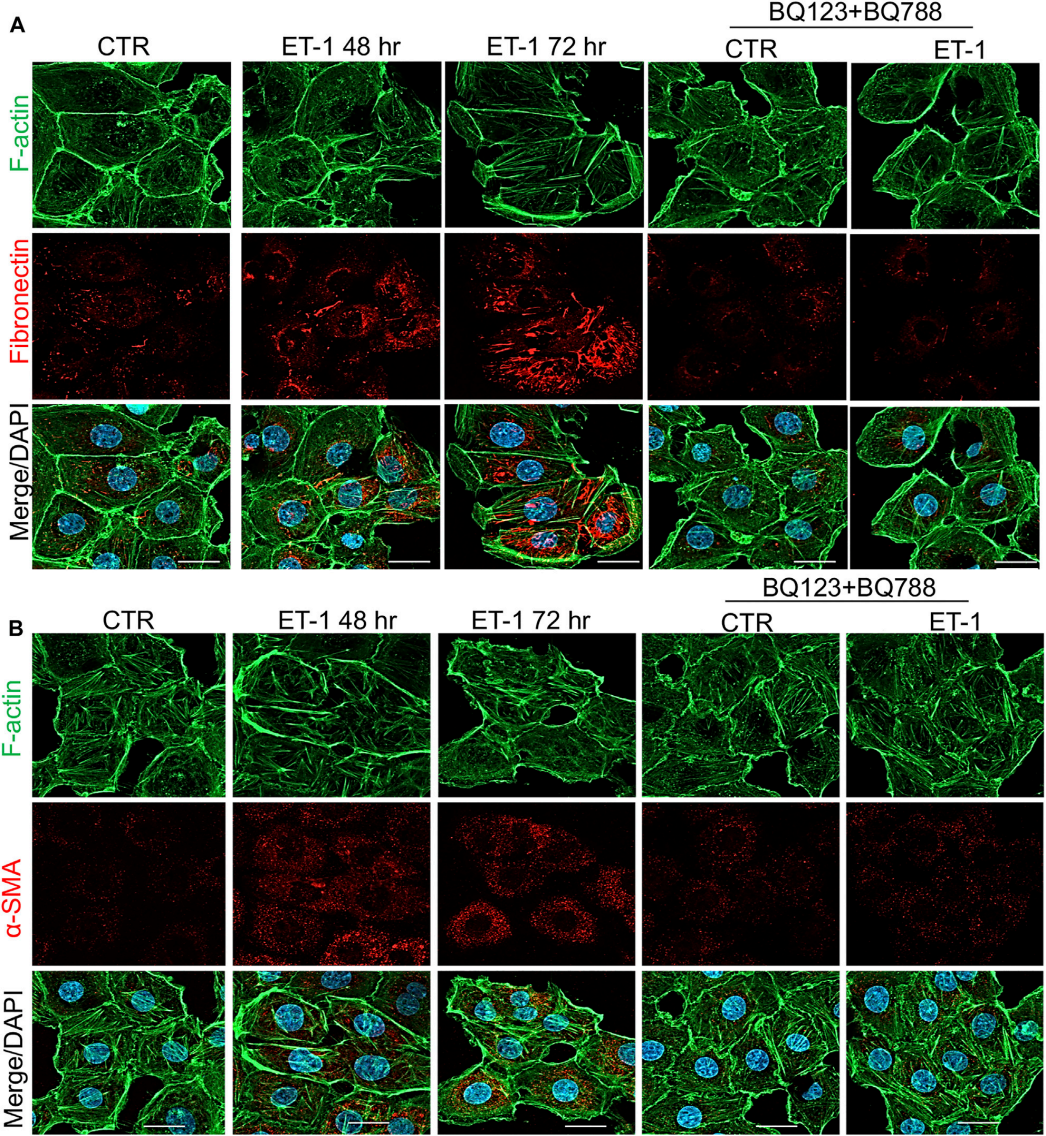
ET-1 upregulates Fibronectin and α-SMA expression through ET_A_R/ET_B_R. Representative images of IF analysis of MCs stimulated with ET-1 and/or BQ123+BQ788 for 48 h. **(A)** Cells are stained for F-actin (green), Fibronectin (red), and DAPI (blue) or **(B)** for F-Actin (green), α-SMA (red) and DAPI (blue). Bars, 20 µm.

To further evaluate the mesenchymal conversion of the MCs, the expression of E-cadherin, TJ protein ZO-1, Fibronectin and α-SMA, another mesenchymal marker, were also examined by IF. MCs show a high membrane E-cadherin and ZO-1 expression (Figures 4A,B) and low α-SMA and Fibronectin expression (Figures 5A,B). Treatment with ET-1 between 48 and 72 h induces a loss of plasma membrane E-cadherin and ZO-1 and upregulates expression of cytosolic α-SMA and Fibronectin, while these effects are reverted in cells treated with both receptor antagonists (Figures 4A,B, 5A,B).

**FIGURE 6:**
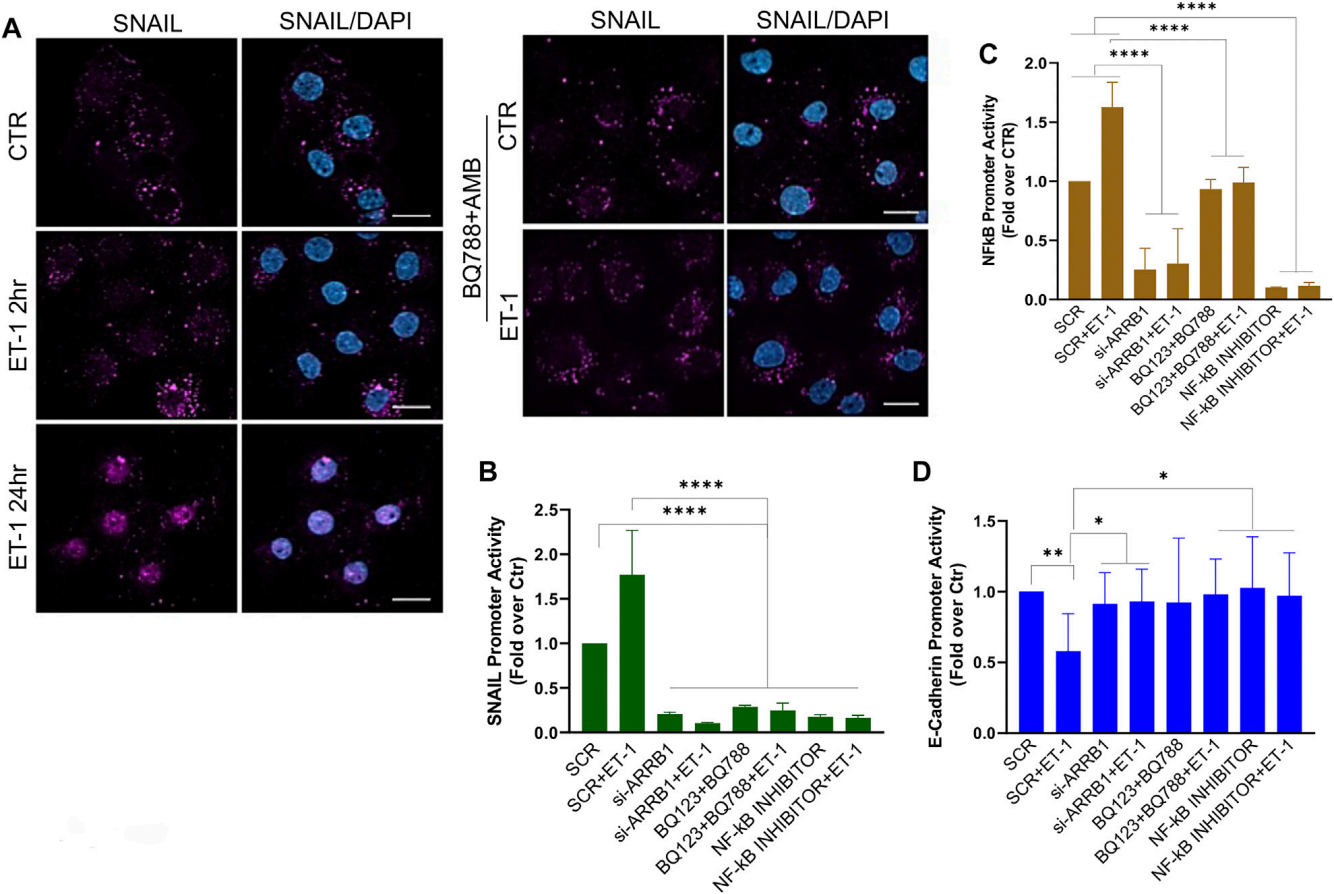
ET-1 regulates MMT through NF-κB/Snail transcription activity. **(A)** Representative images of IF analysis of MCs stimulated with ET-1 and/or BQ788+AMB 24 h and stained for Snail (violet) and DAPI (blu). si-SCR or si-ARRB1-transfected MCs stimulated with ET-1 and/or BQ123+BQ788 or NF-κB inhibitor for 24 h **(B)** SNAIL promoter activity analysis or **(C)** NF-κB promoter activity or **(D)** E-cadherin promoter was calculated as Firefly Luc value/ GAL enzyme activity and shown as fold-over CTR. Histograms, the mean ± SD, *n* = 6. One-way ANOVA and shown as fold-over CTR.

To dissect the molecular mechanisms controlling MMT we focused on Snail, one of the master EMT-related transcription factors (Barberà et al., 2004). IF experiments show increased nuclear staining of Snail in MCs after treatment with ET-1, while this effect is lost upon pre-treatment with BQ788 and AMB (Figure 6A). Then, we examined Snail promoter activity and found that ET-1 induces a significant increase in its activity, which remains unchanged when cells are pre-treated with both BQ123 and BQ788 or after β-arr1 silencing (Figure 6B). Since activation of NF-kB is related to an aggressive phenotype, plays a crucial role in EMT induction in different cell types including MCs, and its target proteins include Snail (Barberà et al., 2004), we evaluated whether ET-1-dependent NF-κB signaling might be involved in MMT occurrence. By using an NF-kB luciferase reporter vector, we found an upregulated NF-kB signaling promoter activity in the ET-1 cultured cells but not in cells cultured in the presence of BQ123+BQ788 (Figure 6C) or β-arr1-silenced cells (Figure 6C), confirming previous data on the role of β-arr1 in the NF-kB pathway (Cianfrocca et al., 2014). Moreover, the addition of the NF-κB inhibitor significantly impairs Snail promoter activity (Figure 6B). Because Snail is implicated in the repression of E-cadherin transcription (Barberà et al., 2004), we analyzed E-cadherin promoter activity. ET-1 suppresses the transcriptional activity of E-cadherin through both receptors, as indicated by the inhibitory effect of both BQ123 and BQ788 (Figure 6D). A similar effect is observed upon silencing of β-arr1 as well as by the NF-kB inhibitor (Figure 6D).

These data strongly support the idea that an ET-1/β-arr1/NF-kB signaling sustains a mesenchymal gene program leading to MMT.

### ET-1 Confers Migratory and Invasive Phenotype to MCs

**FIGURE 7:**
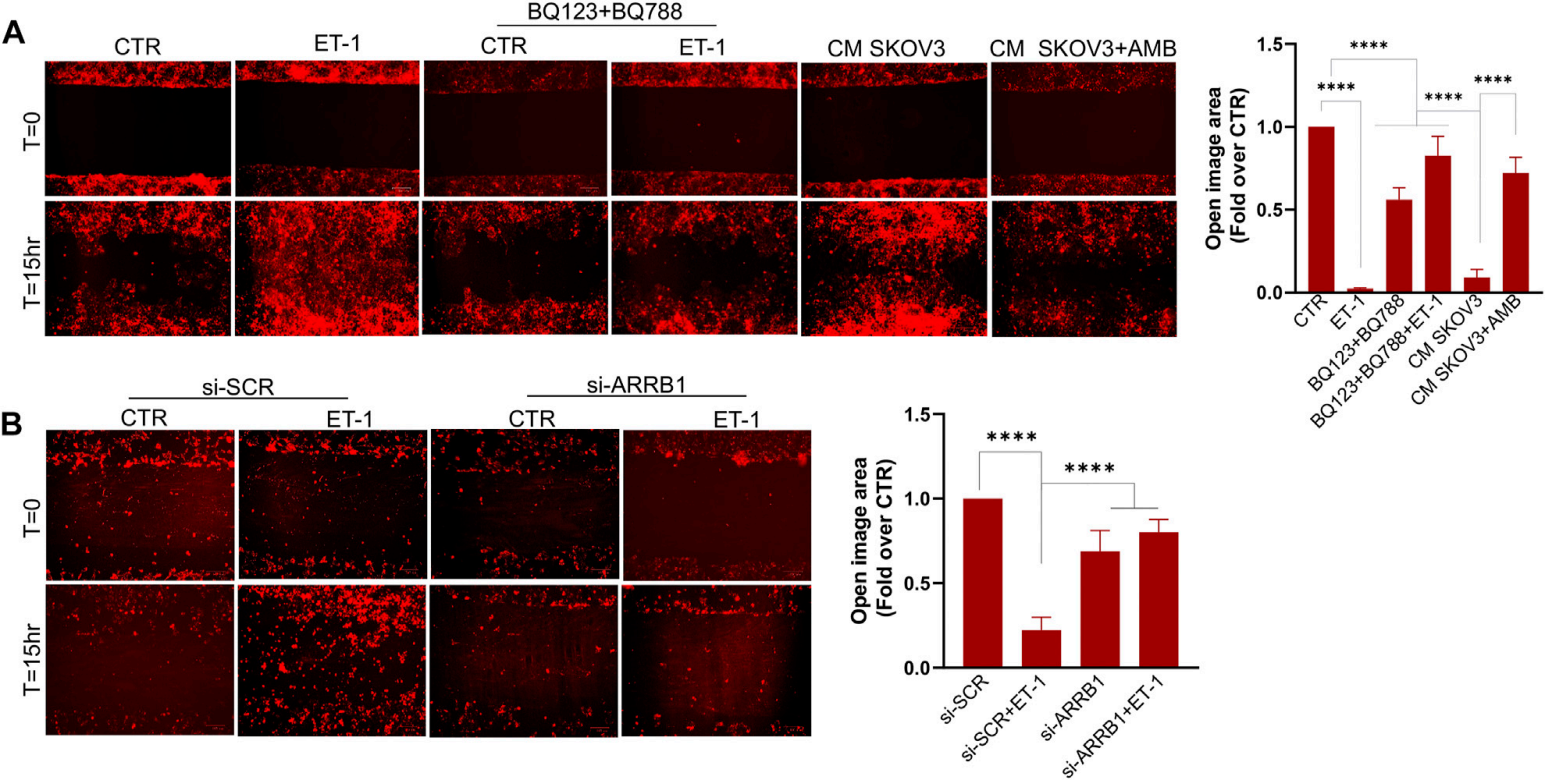
ET-1 promotes migration of MCs through the ET_A_R/ET_B_R/β-arr1 axis. **(A)** Wound healing assay in MCs after addition of ET-1 and/or BQ123+BQ788 or CM from control or AMB-treated SKOV3 for 15 h. **(B)** Wound healing assay in MCs transfected with si-SCR and si-ARRB1 and stimulated by ET-1 for 15 h. Histograms, the mean ± SD, *n* = 6. One-way ANOVA and shown as fold-over CTR.

We next investigated whether ET-1 promotes MCs motility. As shown by wound-healing assays, ET-1 promotes MC migratory potential, reaching the almost complete wound closure after 15 h, but not when cells are pretreated with BQ123 and BQ788 (Figure 7A). As reported for cell proliferation, the addition of CM from control SKOV3 and HEY cells, but not from cells pretreated with AMB, induces a significant wound closure (Figure 7A; Supplementary Figure S2A), demonstrating that ET-1 derived from SOC cells might promote cell migration. Of note, significant inhibition of ET-1 effect is observed in MCs silenced for β-arr1 compared with SCR-transfected cells (Figure 7B), indicating the involvement of β-arr1 in this process.

**FIGURE 8:**
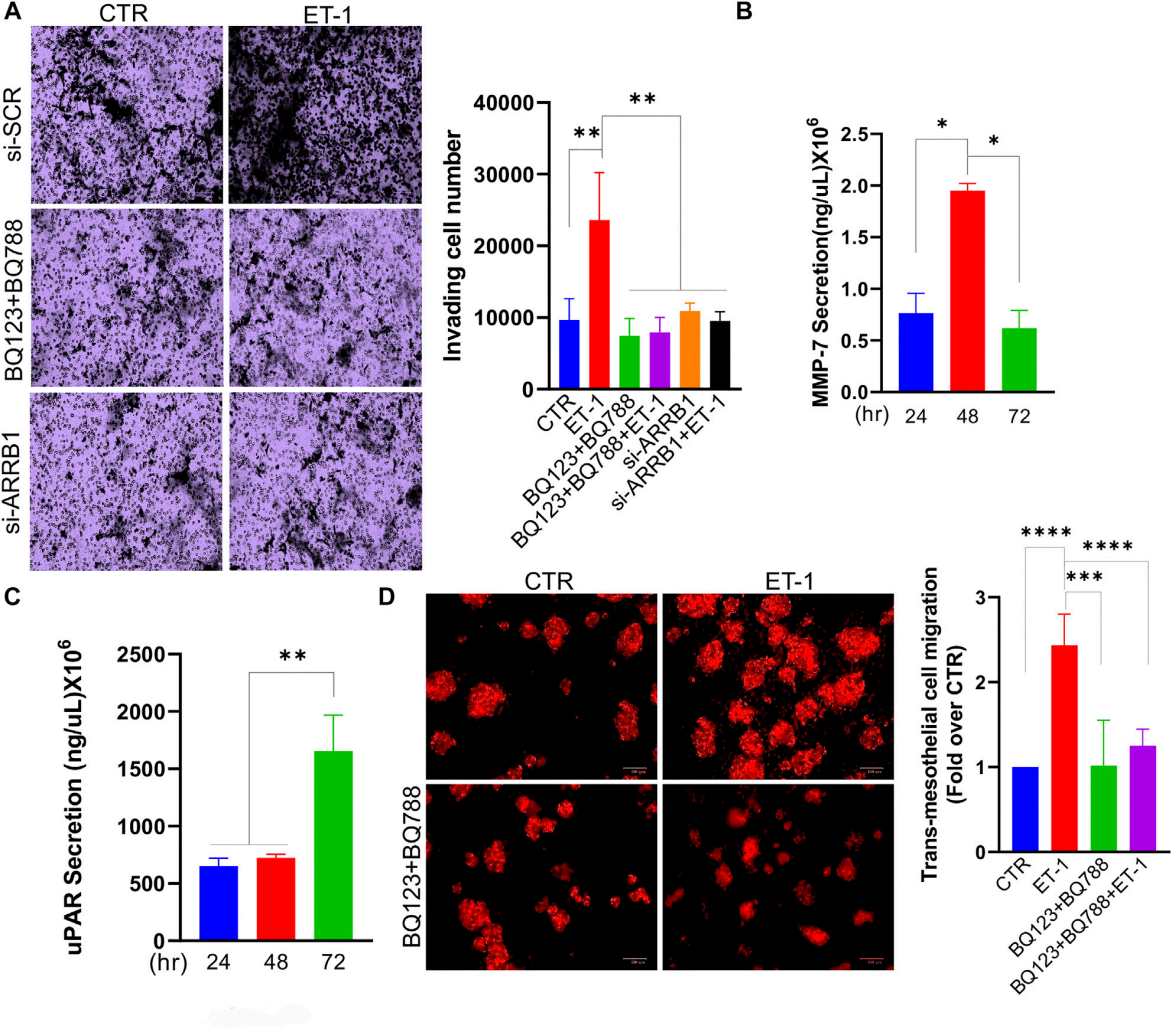
ET-1 promotes invasion of MCs and transmesothelial migration of SOC cells. **(A)** Transwell invasion assays of MCs transfected with si-SCR or si-ARRB1 and stimulated with ET-1 and/or BQ123+BQ788 for 12 h. Histograms, the mean ± SD, *n* = 6. One-way ANOVA. **(B)** MMP-7 or **(C)** uPAR secretion evaluated by ELISA in CM from MCs at indicated times. Histograms, the mean ± SD, *n* = 2. One-way ANOVA. **(D)** Transmesothelial migration assay of OVCAR3 cells seeded into the upper chamber with a monolayer of MCs on a fibronectin-coated membrane and treated with ET-1 and/or BQ123+BQ788 for 24 h. Histograms, the mean ± SD, *n* = 6. One-way ANOVA.

We also tested the invasive behaviour of MCs upon ET-1 addition and CM from SOC cells, by transwell invasion assays. Significant induction of invasive activities by ET-1 is inhibited by BQ123 and BQ788 pre-treatment or after β-arr1 silencing (Figure 8A). The invasive effect of CM from SKOV3 and HEY cells is inhibited by pre-treatment with AMB (Supplementary Figure S2B). In agreement with these results, enhanced secretion of tumor-related proteases such as MMP-7 and uPAR, are observed in ET-1-treated MCs (Figures 8B,C).

We previously demonstrated that the ET-1/ET_A_R axis in SOC cells facilitates adhesion and transmesothelial migration (Masi et al., 2021a). To investigate whether ET-1-induced MMT conversion might impact the transmesothelial migration of SOC cells, we used a co-culture system using fluorescent-labelled OVCAR3 cells and MCs previously cultured with or without ET-1 and/or BQ123 and BQ788. Compared to cells transmigrating through meso-mimetic cultures of control MCs, significantly more tumor cells migrate through MCs cultured with ET-1 but not with BQ123 and BQ788 (Figure 8D), supporting the role of ET-1-dependent signaling in MCs to facilitate the communication between SOC and mesothelial cells, and cell invasion.

### The ET-1 Receptors are Co-Expressed With Mesothelial-to-Mesenchymal Transition Markers in Human Serous Ovarian Cancer Peritoneal Implants

**FIGURE 9:**
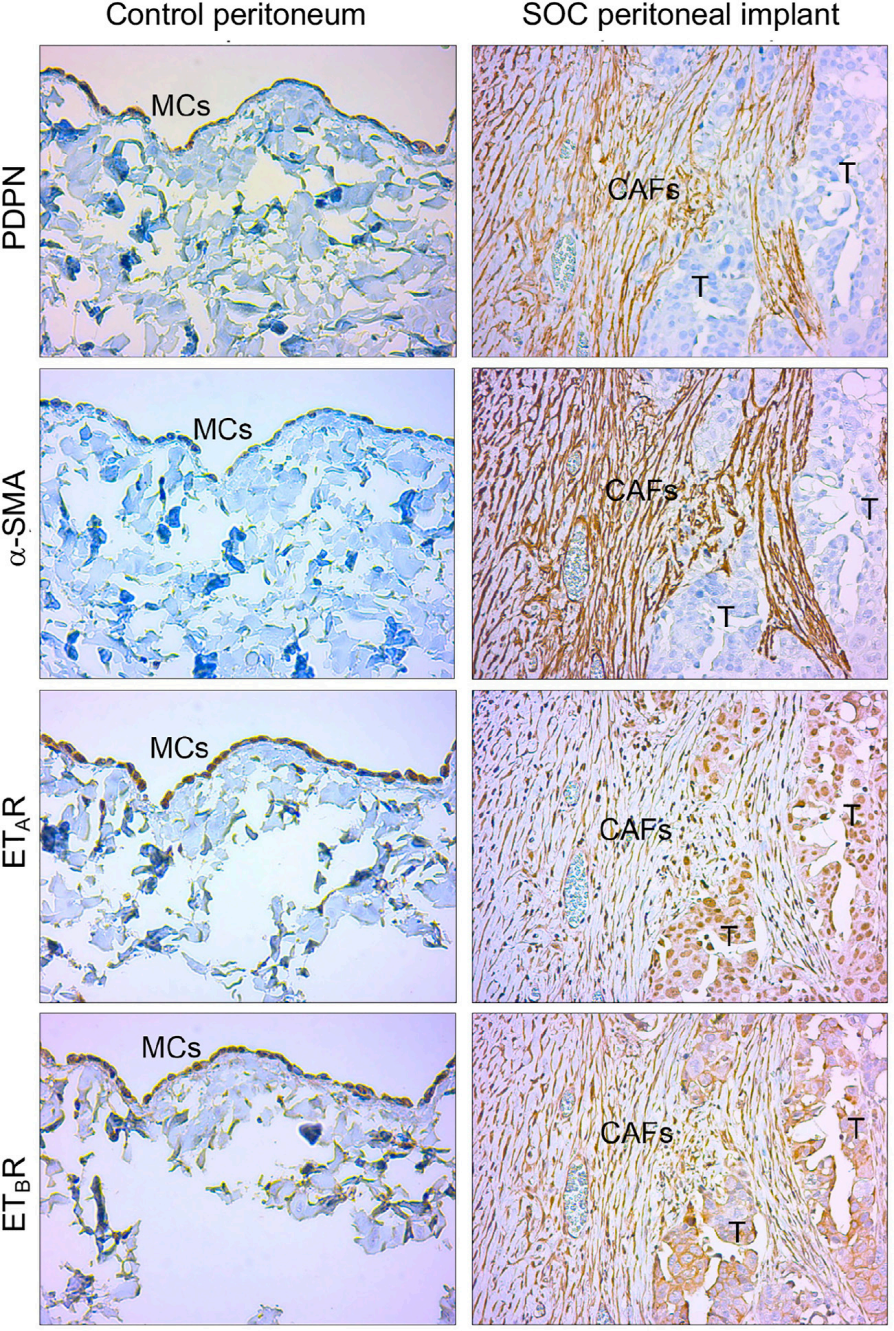
ET-1 receptors are co-expressed with MMT markers in human SOC peritoneal implants. Representative images of IHC analysis of control peritoneum and SOC peritoneal implant sections, stained for ET-1 receptors (ET_A_R, ET_B_R) and MMT markers (PDPN and α-SMA).

To enhance the translational relevance of our findings, we took advantage of IHC analysis of peritoneal biopsies from normal and SOC tissues for evaluating the expression of ET-1 receptors with MMT markers. Immunostaining of serial sections showed the presence of fibroblast-like cells expressing α-SMA and mesothelial marker podoplanin (PDPN) in the submesothelial area of peritoneal implants and the co-expression of both ET_A_R and ET_B_R (Figure 9), confirming the hypothesis that in peritoneal metastases fibroblast-like cells might derive from MCs via ET-1 receptor-dependent MMT. In a control peritoneum, there was a basal expression of ET-1 receptors, as expected.

## Discussion

**FIGURE 10:**
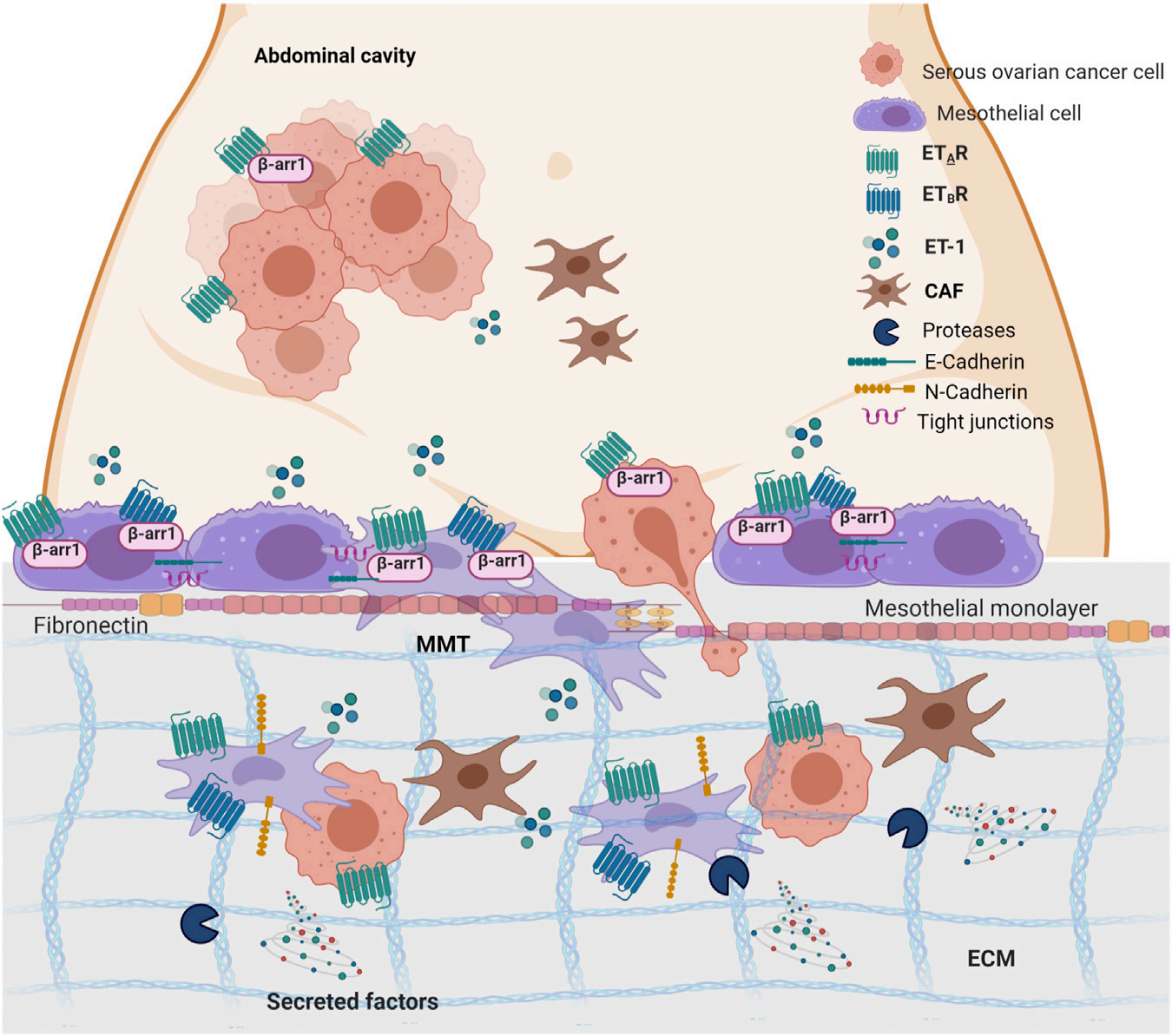
ET-1 promotes mesothelial to mesenchymal transition (MMT) through ET_A_R/ET_B_R/β-arr1 axis. The working model describes the crosstalk between MCs and SOC cells. Autocrine and paracrine ET-1 activates ET_A_R and ET_B_R in mesothelial cells leading to MMT, migration and invasion and favouring transmesothelial migration of SOC cells. Created with BioRender.com.

The progression of SOC cells to metastasis relies on the dynamic communication between cancer cells and their TME, highlighting the importance of our understanding of how communication signaling sent by cancer cells are interpreted and translated into stromal cells to improve the diagnosis and prognosis of this tumor (Naora and Montell, 2005). In the abdominal cavity, the peritoneum/omentum offers a highly compatible microenvironment for SOC cell metastasis, where tumor cells might alter the host environment into a favorable peritoneal seed. The emerging concept is that the functional interaction between cancer and MCs supports the first step of metastatic colonization. Moreover, tumor-derived bioactive substances might foster MC reprogramming in cancer-associated MCs, similarly to that observed for cancer-associated fibroblasts and adipocytes (Aziz et al., 2019). In this study, we provide evidence into the cellular and molecular features linking ET-1 to cancer-associated MCs (Figure 10). Mechanistically, we demonstrated that 1) MCs express both ET_A_ and ET_B_ receptors, along with β-arr1, and secrete ET-1; 2) An autocrine/paracrine ET-1 loop drives p42/44MAPK and NF-kB pathways supporting cell proliferation; 3) ET-1 receptors/β-arr1 activation elicits an MMT program, triggering the enhanced secretion of cancer-related proteins, MC migration and invasion, and SOC cell transmesothelial migration; 4) In SOC peritoneal specimens, ET-1 receptors co-localize with MMT markers.

Besides contact-mediated signaling and direct mechanical interactions, cancer-derived soluble growth factors and cytokines might foster changes in the cellular and molecular features of non-cancerous cells, enhancing the malignant properties of cancer cells (Aziz et al., 2019). In this context, previous studies strongly supported ET-1, present in the ascitic fluid of SOC patients and secreted by stromal and cancer cells, as a key factor for tumor-associated stromal cells (Rosano’ et al., 2013). Our data show for the first time MCs as a new source for ET-1 in the ovarian cancer TME, which can act as an autocrine factor and can cooperate with tumor-derived ET-1, to drive MC proliferation and cancer-associated MC behaviour. Because of this ET-1 loop, MCs acquire the capacity to secrete inflammatory, pro-invasive and angiogenic factors, supporting the growth and motility of cancer cells.

Our recent data demonstrated the existence of a signaling pathway driven by ET-1/ET_A_R and converging on ILK in facilitating the interaction of SOC cells with MCs. Through this pathway, cancer cells might adhere and bypass the mesothelial barrier via invadopodium formation (Masi et al., 2021b). Results from this study suggest that autocrine/paracrine ET-1 in MCs might provide a more conducive environment for SOC cells to undergo invasion. The new perspective is that, upon ET-1 axis activation in cancer and stromal cells, the aggressive SOC cells might perpetuate within the stromal compartments populated with active and reactive cells, like cancer-associated MCs, which are integrated into the tumor architecture favouring their pro-metastatic functions.

Although the importance of the mesenchymal program in SOC ability to invade and clear the mesothelial layer (Davidowitz et al., 2014; Mogi et al., 2021), a current working model suggests a similar genetic reprogramming, MMT, by which MCs change morphology, taking on a fibroblastic rather than epithelial-like appearance in the metastatic niche. This complex process supports adhesion, invasion, vascularization as well as tumor-regrowth, and seems to be determined by cues from the TME rather than just the cellular genome. Peritoneal MCs affected by tumor mediators display features consistent with MMT activation, and a high migratory and invasive potential, all of which are distinctive of reactive MCs. We provide new insights into the ability of tumor and stroma-derived ET-1 to educate MCs to a pro-metastatic function by causing MMT, thus expanding the list of factors implicated in the activation of MCs in SOC (Strippoli et al., 2008; Fujikake et al., 2018; Peng et al., 2019). Our findings are in line with a previous work showing that ET-1 and both receptors were upregulated in the peritoneum exposed to peritoneal dialysis, while ET-1R blockade resulted in a marked attenuation of membrane structural and functional alterations. At molecular level, ET-1 downstream of TGF-β1 acts as an inducer of MMT and fibrocyte recruitment, strongly supporting a role for ET-1 as a contributor of peritoneal fibrosis in mesothelial cells (Busnadiego et al., 2015). We also provide new evidence on the role of β-arr1 in these stromal cells, regulating pathways linked to cell proliferation and MMT. The induction of cancer-associated MCs by ET-1/β-arr1 involves at least two steps; the first via priming MCs through a secretory phenotype, and the second through the maturation of carcinoma-associated fibroblast (CAF)-like phenotype via NF-kB/Snail signaling. In this context, a previous study reported that β-arr1 is expressed in stromal cells and acts as a mediator of fibroblast motility, invasion and cell shape, which can be inhibited by targeting β-arr1 by a small molecule (Suvarna et al., 2018).

As our understanding of the biology of ovarian TME is evolving, new therapeutic strategies by interrupting tumor/MC bidirectional signaling are emerging. Very powerful studies deconstructing and then reconstructing high-grade SOC omental metastasis, to develop multicellular 3D models and dissect the role of cancer/stroma interaction in early stages of metastatic disease, further confirmed that MCs become activated similarly to malignant cells and a significantly high expression of genes related to mesenchymal transformation is upregulated in malignant cells and MCs (Pearce et al., 2018; Malacrida et al., 2021). On the other hand, cancer-associated MCs might influence in a paracrine manner SOC cell motility not only through secreted soluble factors but also by modifying the stromal ECM, highlighting the importance of targeting MCs to inhibit malignant cell invasion and metastasis (Iwanicki et al., 2011; Davidowitz et al., 2014; Natarajan et al., 2019). Although further analyses are required to dissect the crosstalk between cancer and mesothelial cells driven by ET-1/β-arr1 in new multicellular models, which replicate more closely the disease tissues and identify new proteins involved in the activation of cancer-associated MCs, we speculate that targeting the ET-1 signaling could also interfere with the accumulation of cancer-associated MCs and with tumor colonization through the peritoneum. Moreover, new *in vivo* studies targeting this crosstalk by interrupting the ET-1/β-arr1 pathway might give us insights into the development of targeted therapies against MC-derived CAFs, which occupy a major portion of SOC TME.

## Data Availability

The raw data supporting the conclusion of this article will be made available by the authors, without undue reservation.
