# Supplementary figures and images for: Ovarian Cancer-Driven Mesothelial-to-Mesenchymal Transition is Triggered by the Endothelin-1/β-arr1 Axis
Source: Front Cell Dev Biol. 2021 Dec 1;9:764375. doi: 10.3389/fcell.2021.764375 (PMC8672058; doi:10.3389/fcell.2021.764375)

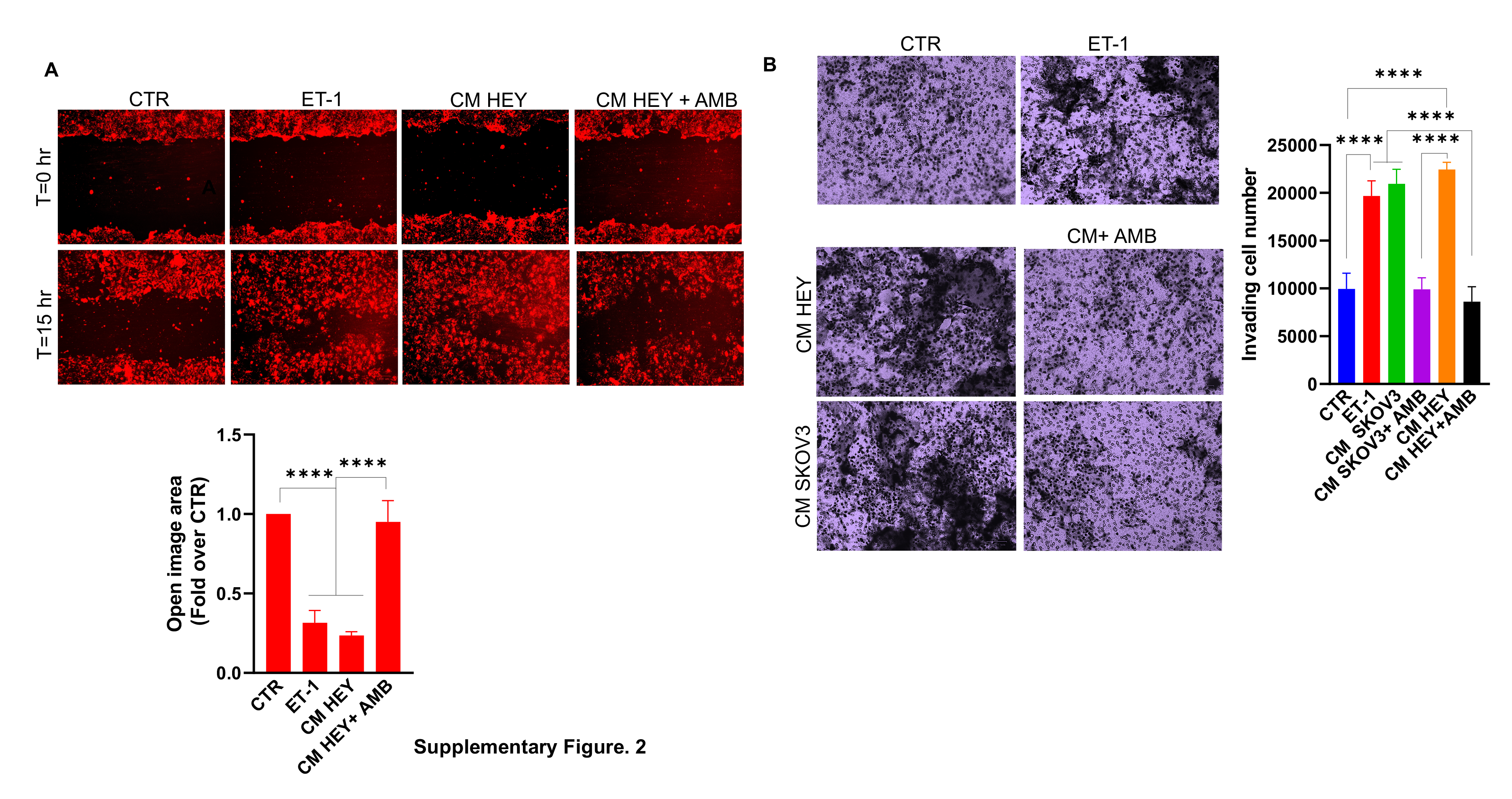

Supplement: Supplementary file 1 [file Image2.TIF]

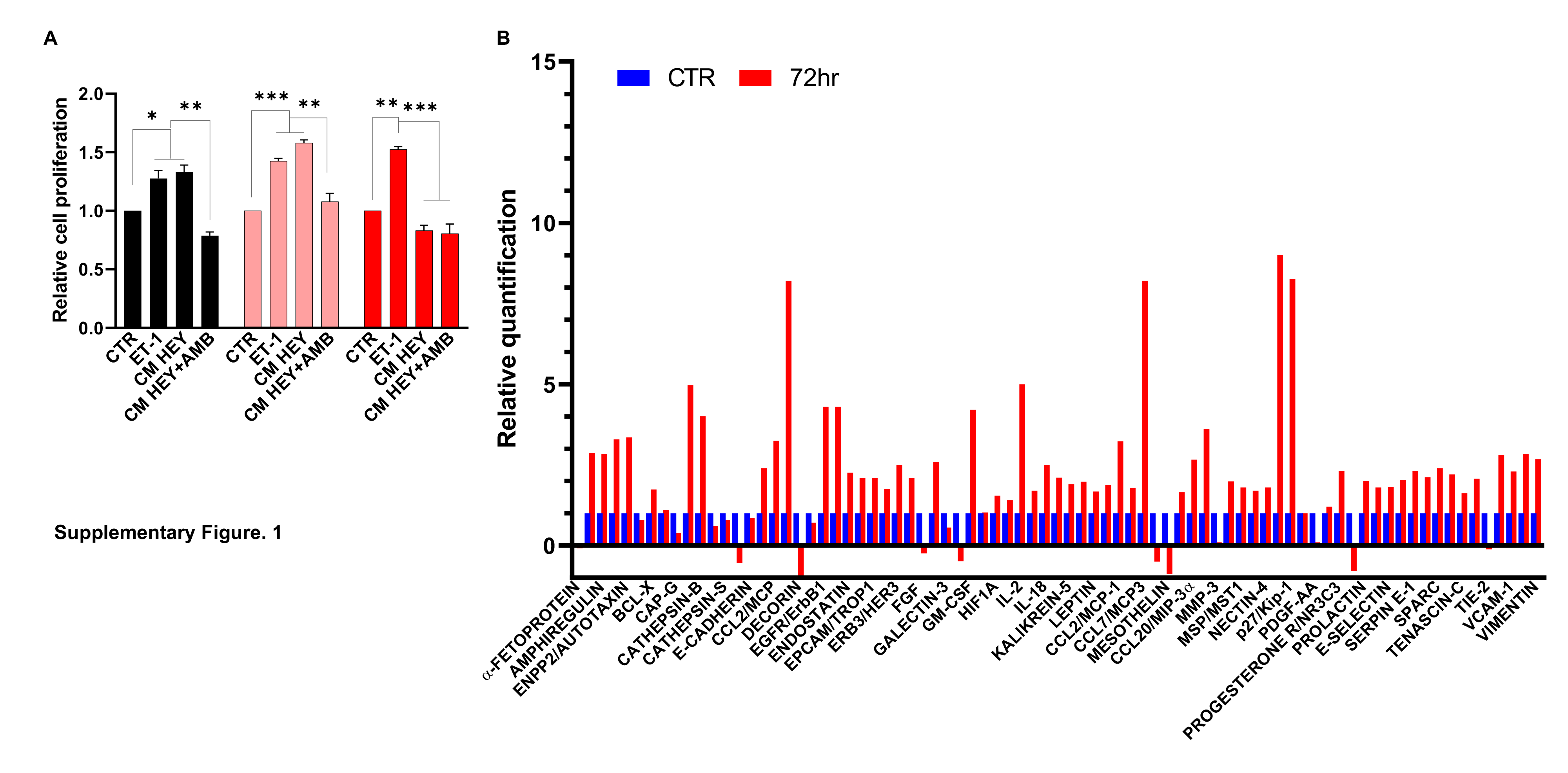

Supplement: Supplementary file 2 [file Image1.TIF]
